# Revolutionize livestock breeding in the future: an animal embryo-stem cell breeding system in a dish

**DOI:** 10.1186/s40104-018-0304-7

**Published:** 2018-12-14

**Authors:** Zhuocheng Hou, Lei An, Jianyong Han, Ye Yuan, Dongbao Chen, Jianhui Tian

**Affiliations:** 1 Key Laboratory of Animal Genetics, Breeding and Reproduction of the Ministry of Agriculture, National Engineering Laboratory for Animal Breeding, College of Animal Science and Technology, China Agricultural University, Beijing, China; 2 State Key Laboratories for Agrobiotechnology, College of Biological Sciences, China Agricultural University, Beijing, China; 3 Colorado Center for Reproductive Medicine, Denver, USA; 4 Department of Obstetrics and Gynecology, University of California Irvine, Irvine, USA

**Keywords:** Animal breeding, Embryos, Genomic selection, In vitro germ cell induction, Pluripotent stem cells

## Abstract

Meat and milk production needs to increase ~ 70–80% relative to its current levels for satisfying the human needs in 2050. However, it is impossible to achieve such genetic gain by conventional animal breeding systems. Based on recent advances with regard to in vitro induction of germ cell from pluripotent stem cells, herein we propose a novel embryo-stem cell breeding system. Distinct from the conventional breeding system in farm animals that involves selecting and mating individuals, the novel breeding system completes breeding cycles from parental to offspring embryos directly by selecting and mating embryos in a dish. In comparison to the conventional dairy breeding scheme, this system can rapidly achieve 30–40 times more genetic gain by significantly shortening generation interval and enhancing selection intensity. However, several major obstacles must be overcome before we can fully use this system in livestock breeding, which include derivation and mantaince of pluripotent stem cells in domestic animals, as well as in vitro induction of primordial germ cells, and subsequent haploid gametes. Thus, we also discuss the potential efforts needed in solving the obstacles for application this novel system, and elaborate on their groundbreaking potential in livestock breeding. This novel system would provide a revolutionary animal breeding system by offering an unprecedented opportunity for meeting the fast-growing meat and milk demand of humans.

## Introduction

As the main dietary protein sources, meat and milk production requires approximately 70–80% increase relative to current levels [1, 2] in order to meet the demand of the predicted 9.6 billion human population in 2050 [3, 4]. However, it is difficult to increase meat and milk production by raising more livestock as the global yield of major crops will peak in the near future [5, 6]. In addition, large expansion of livestock head would create an environmental threat because the greenhouse gas emission by livestock accounts for approximately 14.5% of human-induced global emissions [7]. Hence, because of the upper limit of major crop yields and total head of livestock, improving animal feed and production efficiency is the only way to provide enough protein sources for human needs in future. Genetic selection is one of the most important means for improving livestock production [8]. However, genetic improvement in feed conversion efficiency by conventional breeding is very slow during the past decades in farm animals including swine, cattle, sheep, and goats. Annual genetic improvement in feed converion efficiency is estimated to be only 0.7% in swine [9] and this number is even lower in cattle and sheep [10]. Genetic improvement in other important economic traits, e.g., disease resistance and fertility [10], are also slow or even stagnant. Further, more traits are expected to be included as important considerations in future breeding schemes. Thus, the global demand for milk and meat production requires more efficient and sustainable animal breeding systems for accelerating genetic improvement [8, 11].

During the past decades, researchers have made encouraging progresses in improving animal breeding efficiency and have realized many proposed concepts. From 1990’s, several landmark studies [12, 13] concluded that marker-assisted selection (MAS) can improve the animal breeding efficiency. As most economic traits are controlled by multiple genes/alleles, single MAS cannot be effectively applied in animal breeding, that is why genomic selection methods are needed for improving selection accuracy. After long-term MAS theoretic studies, genomic selection (GS) was first coined in 1998 [14], and later genomic selection theoretic framework was proposed in 2001 [15]. With the help of quick progresses in high-density chip and high-throughput sequencing, genomic selection was first used in dairy breeding after 10 years of proposing genomic selection concept [16]. Until now, GS has been widely implemented in swine, beef cattle, and chicken breeding. Threrefore, it takes more than 20 years from the conceiving MAS concept to large-scale industrial application of GS. In addition, the combination of embryonic technologies and MAS was also proposed to improve animal breeding efficiency [14]. As early as 1980–1990s, it has been recognized that embryonic technoloies such as oocyte pick-up (OPU), in vitro fertilization (IVF), and preimplantation genetic diagnosis (PGD) could be potentially applied to intensify breeding process. Due to the continuous improvement of efficiency in these molecular and embryonic technologies, many of these conceptions have been achieved or even industrially applied in animal breeding.

Recent advances in stem cell biology offer an unprecedented opportunity for revolutionizing the animal breeding system. Using pluripotent stem cells (PSCs), including both embryonic stem cells (ESCs) and induced pluripotent stem cells (iPSCs), germ cells can be induced in vitro to complete the entire gametogenesis processes and form functional spermatids or oocytes [17, 18]. In natural conditions, in vivo gametogenesis need to go through both fetal and postnatal gonadal development, which usually take several months to over 1 year in large farm animals. However, using mouse as model, in vitro induction of germ cells can reconstitute gametogenesis in a much shorter time (e.g. 6 weeks in mice). Moreover, embryos can be obtained from in vitro generated spermatids and oocytes, and then the blastocysts can be further used to derive ESCs, which is designated as regenerated ESCs (rESCs). The rESCs can subsequently undergo complete gametogenesis via a new round of in vitro germline induction [17].

**Fig. 1:**
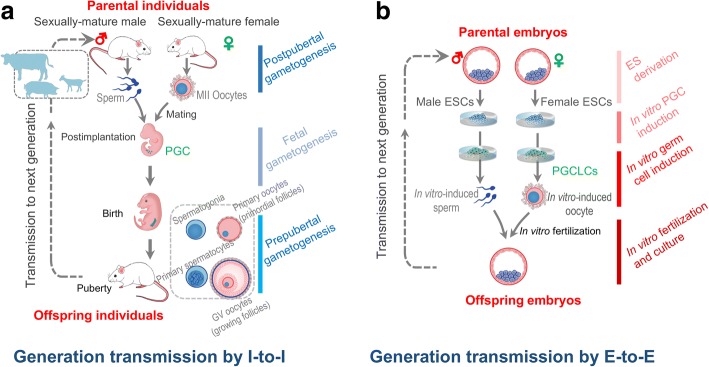
Mammalian generation transmission by parental individuals to offspring individuals (I-to-I) and parental embryos to offspring embryos (E-to-E). **a** I-to-I transmission: gametogenesis is a long-term process highly associated with individual development and growth. PGCs, from which both oocytes and sperm originate, are established by the post-implantation stage. The subsequent oogenesis and spermatogenesis necessarily depend on fetal development and postnatal gonadal growth from birth until puberty. Gametogenesis ensures the creation of new individuals of the next generation of mammals, where genetic information is transmitted to next generation. **b** E-to-E transmission: gametogenesis is induced in vitro in ESCs to form functional oocytes and sperm. The entire process depends on in vivo fetal development and prepubertal growth. The induced oocytes and sperm develop into normal offspring embryos following IVF. The IVF offspring embryos are further used to derive ESCs, which can in turn undergo complete gametogenesis via a new round of in vitro germline induction

This renewed in vitro life cycle perpetuates the direct cross-generational transmission of genetic information from parental embryos to offspring embryos (E-to-E), distinct from the natural cross-generational transmission from parental individuals to offspring individuals (I-to-I) (Fig. 1a). In vitro germline induction, together with subsequent in vitro fertilization (IVF) and ESC derivation, has successfully created new individuals and alternation of generations, thereby reconstituting an entire mammalian life cycle in vitro (Fig. 1b). This rapidly renewed life cycle can be used to constitute a recurrent animal breeding cycle by selecting and mating embryos directly, i.e., IVF using PSC-derived gametes. Thus, we propose a novel animal breeding system termed animal embryo-stem cell breeding system that can revolutionize the design and implementation of current breeding programs in livestock. Here we describe the workflow of the breeding system in view of relevant technologies involved and discuss the challenges and its promising implications.

## Animal embryo-stem cell breeding system

The animal embryo-stem cell breeding system completes a livestock breeding scheme in a dish by integrating in vitro germ cell induction, IVF, genome sequencing, and genomic selection. Based on the in vitro reconstituted life cycles, an animal breeding cycle can be renewed by directly selecting and mating embryos rather than adult individuals, thereby achieving rapid genetic improvement of important economic traits.

### Major procedures

#### Step 1: Form a breeding plan and establish a nuclear breeding population

**Fig. 2:**
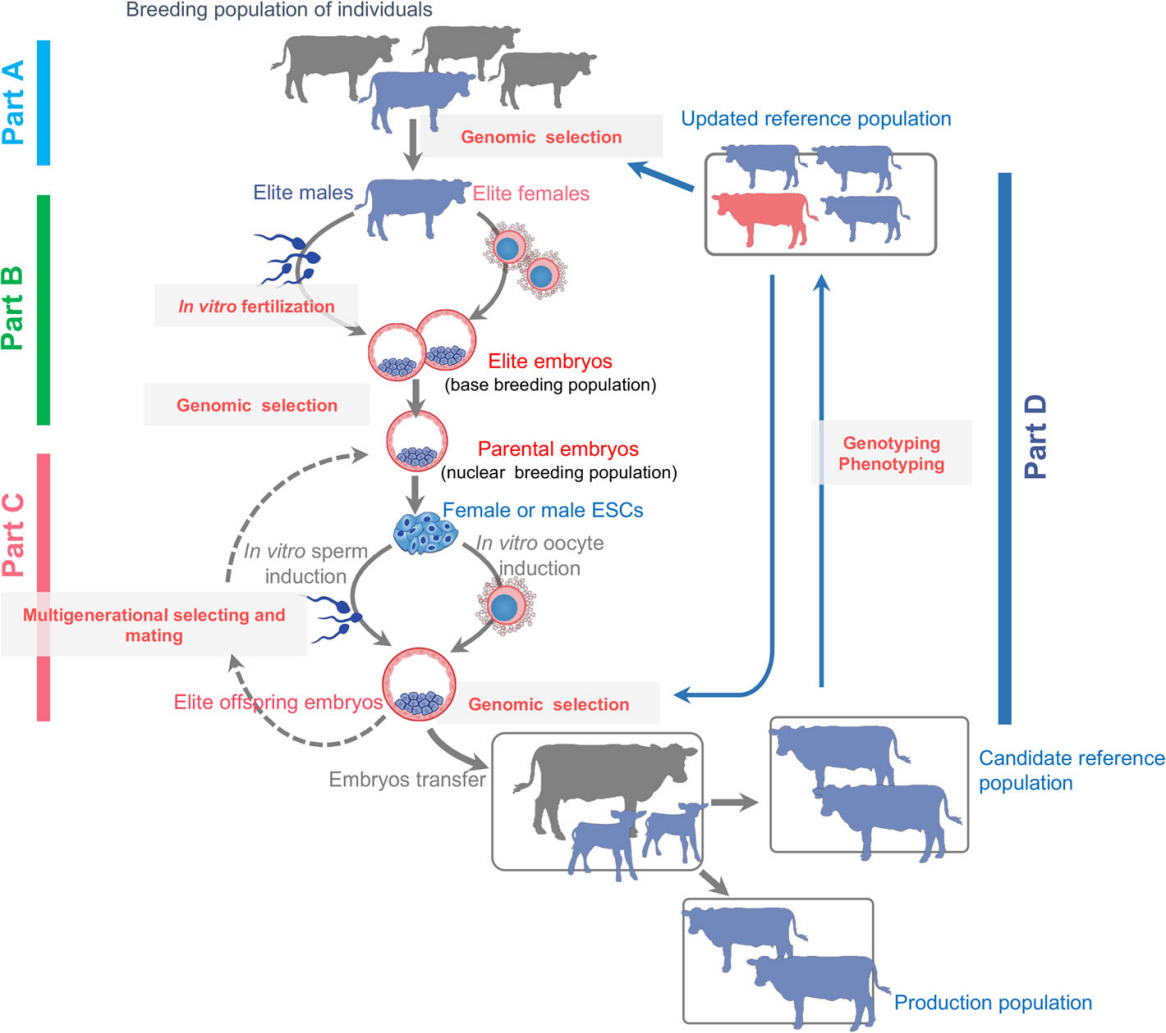
A schematic workflow of the animal embryo-stem cell breeding system**.** The novel system comprises the following modules: Part A:Form a breeding plan and establish a nuclear breeding population; Part B:Establish the base and nuclear breeding population of elite embryos; Part C:Transgenerational breeding cycle from parental embryos to offspring embryos; Part D:Reference population construction and updating

Similar to the conventional breeding system, the stem cell-embryo breeding system also needs to first create a breeding scheme based on market demand and genetic resources of the breeding herd and then establish a platform for genomic selection or use an established platform. The breeding value of each individual should be evaluated and elite candidates will be selected to establish a base breeding population (Fig. 2, Part A).

#### Step 2: Establish the base and nuclear breeding population of elite embryos

Using the sperms and oocytes from individuals with the best breeding values in the breeding population, IVF will be performed to generate male and female base embryos according to the breeding scheme. Genomic estimated breeding values (GEBV) will be evaluated for all base embryos. Embryos with the top GEBV will be used as parental embryos to establish a nuclear breeding population of elite embryos (Fig. 2, Part B).

#### Step 3: Transgenerational breeding cycle from parental embryos to offspring embryos (E-to-E)

This step includes three essential breeding components similar to the conventional breeding system: selective breeding of parental embryos; controlled mating of parental embryos; and multigenerational breeding of embryos.

- Selective breeding of parental embryos: Single cells (blastomeres or trophectoderm cells) will be isolated from parental preimplantation embryos and they will be genotyped by whole-genome sequencing. The genotyping results will be used to predict the embryo genomic estimated breeding value (eGEBV) by which elite parental embryos will be selected as candidates for controlled mating.
- Controlled mating of parental embryos: Selected parental embryos will be used to derive ESCs, which will be subsequently induced in vitro to form sperm or oocytes. Based on the eGEBV-based breeding scheme, in vitro derived gametes will undergo IVF to generate offspring embryos to complete the controlled mating of parental embryos.
- Transgenerational breeding of embryos: Following selective breeding and controlled mating of the parental embryos, the offspring embryos will undergo a new round of selective breeding and controlled mating, which in turn starts a new selection cycle (Fig. 2, Part C). By repeating this process, the embryo-stem cell breeding system can achieve rapid transgenerational breeding. It should be noted that live birth is not a prerequisite for achieving the breeding cycles from parental embryos to offspring embryos.

The generation interval of the embryo-stem breeding system spans from parental embryos to offspring embryos, involving ESC derivation, in vitro germ cell induction, and IVF. It should be mentioned that ESCs are more preferred for constructing transgenerational breeding cycles. In contrast, the use of iPSCs, or germline-potential stem cells, will prolong the breeding cycle because differentiated fetal or adult somatic cells are needed. The entire breeding cycle is independent of the lengthy processes of pregnancy and postnatal growth. Aside from the classic major factors of a conventional breeding system, this system is characterized by direct selection and mating of candidate embryos, followed by an E-to-E breeding cycle, entirely distinct from individual-based conventional breeding selection. A detailed comparison between the embryo-stem cell breeding system and conventional breeding system is summarized in Table 1.

**Table 1:** Comparisons of major elements among different breeding systems

Major breeding elements	Conventional breeding	Genomic selection	Embryo-stem cell breeding
Breeding scheme	Yes	Yes	Yes
Pedigree record	Yes	Yes, can also reconstruct pedigree from genotyping data	Yes, can also reconstruct pedigree from genotyping data
Performance testing	Breeding animals	Only for reference population	Only for reference population
Reference population	No	Yes	Yes
Candidate breeding animal	Individual	Individual, embryo	Embryo
Generation transfer	Individual to individual	Individual to individual	Embryo to embryo
Breeding value	EBV	GEBV	eGEBV
Gametogenesis	In vivo gametogenesis	In vivo gametogenesis	In vitro induced gametogenesis
Fertilization /Embryo	In vivo fertilization and development; In vitro fertilization and culture	In vivo fertilization and development; In vitro fertilization and culture	In vitro fertilization and culture

During the transgenerational breeding of embryos, the reference population can be updated as frequently as needed. In general, individual production performance and genome-wide single-nucleotide polymorphisms (SNPs) are required to construct original reference population under the breeding plan guidelines as discussed in detail previously [19]. The progeny of the elite breeders among the breeding embryo nucleus can be transferred directly to the commercial production population or performance testing population. The original reference population will be updated by new phenotypic data obtained from the performance testing population (Fig. 2, Part D).

### Advantages of the animal embryo-stem cell breeding system

Key factors affecting genetic gain (R*=*$\frac{\mathrm{i}\times \mathrm{h}\times \mathrm{r}{\sigma}_g}{\mathrm{L}}$) include standard deviation of breeding value (*σ*_*g*_), selection intensity (*i*), selection accuracy (*r*), and the generation interval (*L*) [20]. Genomic selection plays an important role in these key factors for accelerating genetic gain [8]. Compared to the conventional breeding method or genomic selection alone, our proposed system has significant advantages in the following aspects*.*

#### Shorter generation interval

The generation interval is about 5–7 years for sire(s) or dam(s) of bulls in the conventional dairy breeding scheme. This can be drastically reduced to approximately 2.5 years by applying genomic selection [16]. However, our proposed E-to-E breeding system will require only approximately 2 months for a complete one generation of selection. The annual genetic gain will increase about 10-fold or even more ($\frac{R_{E-E}}{R_{GS}}=\frac{2.5\times 12}{2}=15\  times$) when compared to the standard dairy genomic selection system if other selection factors are the same. Taking breeding dairy cows as an example, ideally, our envisioned system is expected to be 30–40 times ($\frac{R_{E-E}}{R_{conventiaon}}=\frac{\left(5-7\right)\times 12}{2}=30-40$) more efficient in comparison to the conventional system, meaning that 1-year genetic gain of in vitro breeding can be the same as that of 30–40 years of conventional breeding. However, as the selection limitation and accuracy of genomic selection might decrease over several generations, more theoretical studies are needed.

#### Higher selection intensity

IVF makes it possible to produce 100,000 or 1,000,000 embryos at the same time, which is equivalent to that of 100,000 or 1,000,000 of selected individuals. We can design the best sequencing strategy for genomic selection to achieve the best selection progresses in considering the breeding cost and genetic improvement.

#### Better breeding scheme for monotocous animals

Monotocous animals, such as cows and ewes, naturally produce only a few offspring in its lifetime. The elite females cannot produce enough offspring as needed, even if some IVF technologies can assist females to have more offspring. If we overcome the obstacles in stem cell biology of farm animals and apply them in this system, monotocous females will make much more genetic contribution than conventional breeding program. Thus, the breeding system will introduce more genetic variations to the breeding population, especially for monotocous animals.

#### Easier integration of new biotechnologies

The breeding system provides easy access to the latest technologies for further improvement of the in vitro breeding system because it relies on manipulation of embryos and ESCs that can be performed in a dish. For example, more sophisticated genome editing can be integrated into the system. Harmful mutations within the population can be eliminated via whole-genome sequencing and genome editing. Promotion of alleles by genome editing (PAGE) combined with genomic selection can be 1.08–4.8 times more efficient than genomic selection itself [21].

## Technical basis and challenges

The proposed novel embryo-stem cell breeding system is mainly based on the recently developed technologies for in vitro germ cell induction and the established routines including IVF, genome sequencing, and genomic selection. Recently, functional haploid male and female gametes have been successfully induced in vitro in mice. These works provide a robust paradigm for achieving in vitro germ line induction in farm animals, and could make the proposed breeding system technically feasible, although a series of obstacles need be overcome. A recent study reported that stable bovine ESCs can be efficiently derived from bovine blastocysts, which offers a technical basis for further establishment of in vitro germ cell induction in farm animals [22]. Here, we summarize the current state and recent advances as well as the challenges in supporting this novel breeding system.

## In vitro germ cell induction in mammals

**Fig. 3:**
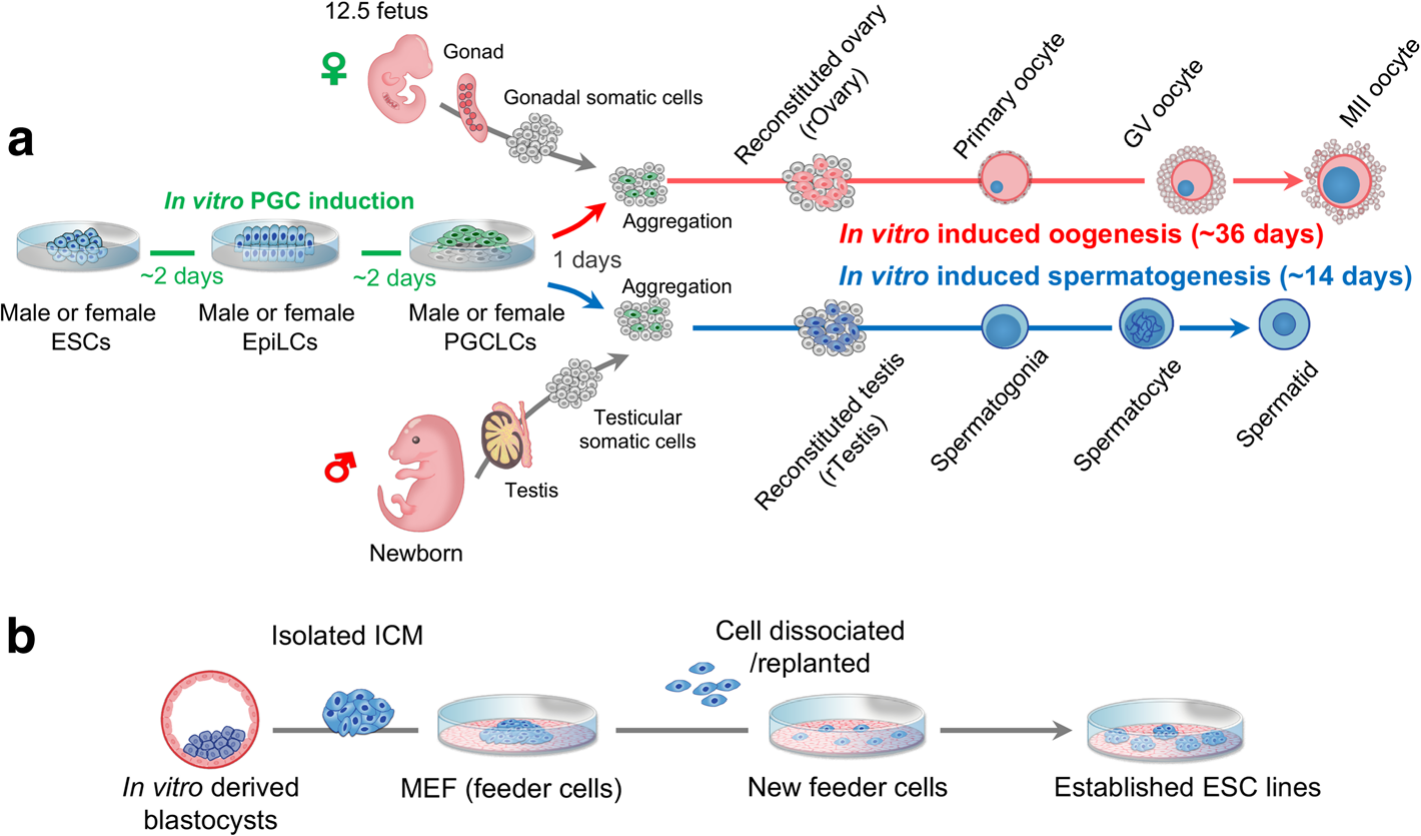
A schematic of ESC derivation and in vitro induced gametogenesis. **a** In vitro induction of functional gametes from ESCs. EpiLCs and PGCLCs are sequentially induced using well-established female or male ESCs. Next, via aggregation with fetal or neonatal gonadal somatic cells under in vitro conditions, in vitro–derived PGCLCs are successfully converted into primary spermatocytes/oocytes respectively, which are further induced into functional haploid sperm and oocytes. **b** Derivation and establishment of pluripotent ESC lines from inner cellular mass (ICM) frim in vitro cultured blastocysts

Until now, using mouse PSCs, the entire germline cycle can be reconstituted in vitro to form functional gametes, although the efficiency remains limited [17, 18]. The generation of primordial germ cells (PGCs), which can initiate meiosis, is of prime importance for generating haploid gametes [23]. Using ESCs bearing the PGC markers PR/SET domain 1 (*Prdm1*, also known as *Blimp1*) and developmental pluripotency–associated 3 (*Dppa3*, also known *Stella*), Hayashi et al reported that the combination of bone morphogenetic protein 4, leukemia inhibitory factor (LIF, interleukin 6 family cytokine) and stem cell factor are highly competent for inducing PGC marker expression in epiblast-like cells (EpiLCs); these cells in turn become PGC-like cells (PGCLCs) to facilitate in vitro induction of PGCs (Fig. 3a). This work provides a robust paradigm for the first step for in vitro gametogenesis. Upon transplantation into an environment of appropriate somatic cells in vivo*,* the induced PGCLCs undergo meiosis and produce functional spermatids and oocytes, which can be subsequently used for generating normal offspring following IVF [24, 25].

More recently, in vitro germ cell induction systems have been further optimized to make meiotic differentiation no longer depend on in vivo gonadal niches. Through aggregation with fetal or neonatal gonadal somatic cells under in vitro conditions, in vitro derived PGCLCs are successfully converted into primary spermatocytes/oocytes, respectively, which can be further induced into functional haploid spermatids and oocytes (Fig. 3a). The functionality of these in vitro derived haploid gametes has been confirmed by the production of viable and fertile offspring via intracytoplasmic sperm injection (ICSI) or IVF [17, 18]. It should be noted that blastocysts derived from the in vitro generated gametes can be further used to derive rESCs, which can undergo a new round of in vitro germline induction. Therefore, by integrating in vitro germ cell induction, IVF, and ESC derivation in mouse models, these studies have successfully reconstituted a recurrent life cycle from parental embryos to offspring embryos, without producing offspring animals [17].

The most prominent challenge for establishing in vitro germ cell induction system in farm mammals may be the pluripotent status of PSCs. Pluripotent ESCs are well-established in mice, rhesus monkeys, and humans (Fig. 3b). However, despite the lengthy history of efforts to establish truly undifferentiated ESCs in farm animals, authentic ESC lines that can be proven by stringent germline chimera assay have not been established conclusively in any of these species. Even using the conditions for generating mouse ESCs, such as LIF, BMP4, inhibitors of GSK3 and ERK (2i), derivation of such cell lines has been shown to be chanllenging in nonrodents, especially in domesticated species [26]. Up to date, the majority of the morphologically resembling ESC lines derived from bovine and porcine embryos/fetus, inlcuding those recovered from natural conception, IVF or somatic cell nuclear transfer, fail to contribute to chimeras and exhibite only limited differentiation potential [27, 28]. It should be mentioned here that the putative porcine ESC lines maintained on a basal medium supplemented with FBS plus three growth factors, namely FGF2, LIF, and KITLG, are more capable of forming teratomas [29]. Thus, it is promising that a combination of growth factors may considerably benefit the system for deriving and maintaining dometic ECS lines, as revealed by the fact that the self-renewal capcity of porcine ES-like cells are both LIF-dependent and FGF2-dependent [27]. Similarly, combined use of LIF and FGF2 is also beneficial for maintaining the bovine ES-like cells in an undifferentiated state [30, 31]. These researches, on one hand, have drawn attention to the importance of formulating culture conditions that are consistent with the apparent requirement of factors essential for maintining pluripotency of domestic ESCs. In addition, these data indicates that significant modifications of culture conditions may be needed even for those that had previously proved so successful for mouse and human, since the mechanism for capturing pluripotency may be considerably different between rodent and domestic species. More recently, Bogliotti et al. reported successful derivation of stable primed pluripotent ESCs from bovine blastocysts by using fibroblast growth factor 2 (FGF2) and an inhibitor of the canonical Wnt–β-catenin signaling pathway (IWR1) to optimize culture condition [22]. This work is a breakthrough as it overcomes the challenge of establishing high-quality pluripotent livestock ESCs. Until now, precise mechanisms of how signaling pathways control the pluripotent state and early embryo development remains largely elusive in farm animals, and it appears that the essential pathways are considerably distinct from those of rodent species. Bogliotti’s study, shows that combination of FGF supplementation and WNT signaling inhibition, both of which are critical for capturing bovine pluripotency and important for normal preimplantation embryo development in bovines [32, 33], is critical for capturing bovine pluripotency. This fact highlights that exploring the mechanism underlying pluripotency of domestic embryos, will help identify major obstacles that hamper the establishment of true ESC lines in domestic animals. However, even high-quality ESC lines are established in farm animals, the efficient PGC specification pathway and subsequent aggregation with gonadal somatic cells remains challenging.

Except the promising studies in ESCs, iPSCs also provide a practical alternative for successful in vitro germ cell induction. By continuous formulation and optimization of reprogramming factors and medium conditions, primed- or naive-type iPSCs have been successfully derived from porcine and bovine embryonic fibroblast cells or other cell types [34–37]. Using porcine iPSCs as progenitor cells, our group has successfully induced porcine iPSCs to the PGCLCs. Further, xenotransplantation of the PGCLCs into seminiferous tubules of infertile immunodeficient mice can result in immunohistochemically identifiable germ cells [38]. Moreover, with the extensive studies over the past decades that investigate the origins and mechanisms underlying PGC and germ line specification/differentiation in domestic animal, a series of key growth factors (e.g. SCF, LIF, FGF2, BMP4) [39–42] and signaling pathways (Activin/Nodal signaling, redox/apoptotic signaling) [42, 43] have been identified to be implicated in maintaining the survival and self-renewal of domestic PGCs. All these findings will benefit the high-efficient system of domestic PGC induction. Interestinly, a more recent study, using in vitro model of germ cell induction, showed conserved principles of epiblast development for PGC fate among porcine and model animals, although the mechanisms underlying pluripotency networks and early post-implantation development are thought to be divergent among species [44]. In addition, studies highlighting the origins of domestic germline-potential stem cells, provide alternate source of domestic PGSs. Aside for those from developing fetal gonad, stem cells derived from adult bovine and porcine ovaries [45, 46] or fetal porcine skin [47, 48] also exhibit the intrinsic ability to differentiate into PGCLCs or even oocyte-like cells (OLCs). However, these germline-potential stem cells are not preferred in our proposed breeding system, because developmentally advanced stem cells will prolong the breeding cycle since differentiated fetal or adult somatic cells are needed. Considering the big challenge of establishing high-quality ESC lines in domestic animals, iPSCs or germline-potential stem cells, may be feasible alternates for connecting transgenerational breeding cycles. Furthermore, Hayashi’s work also offers a valuable reference for formatting and purifying PGCLCs from ESCs without relevant transgenic markers from domestic animals. Specifially, they identified SSEA1 (stage-specific embryonic antigen) and Integrin β3 as essential surface markers for achieving PGCLC isolation and purification [24]. A more recent study further indicated that epithelial cell adhesion molecule (EpCAM) and integrin α6 are efficient in distinguishing PGCLC following human iPS induction [49]. These advances, together with the studies of germ cell biology in porcine and bovine, provide more substantial basis for eventually achieving in vitro germ cell induction in domestic animals.

From the feasibility perspective, a relative low-frequent but noticeable de novo generation of single-nucleotide variants (SNVs) can be elicited in the proposed breeding system, along with the derivation culture and passage of ESCs, especially by the induced reprogramming of iPSCs [50, 51]. For example, dozens to several hundred de novo SNVs can be detected between generations in ESCs or somatic cells and the mutation rate (approximately 10^− 9^ to 10^− 8^ at global genome level) is more frequent than that from in vivo germline differentiation (approximately 10^− 10^ to 10^− 9^ at global genome level which varies largely based on species, cell types, and culture or induction methods). Although de novo mutations induced by the manipulation of pluripotent cells have minimal contributions to the reference sites of genome selection, the biological significance and potential application as well as the risk of de novo mutations should be re-evaluated based on offspring phenotypes.

## In vitro fertilization in domestic mammals

IVF is the process of creating embryos from oocytes by fertilizing them with sperm cells in a dish. A broader definition of IVF in cattle industry often involves oocytes retrieval from the ovaries, including recovery and in vitro maturation of oocytes, and in vitro fertilization and culture of embryos. The high-efficient IVF methodology is an important component of embryo-stem cell breeding system to support large-scale production of highly competent embryos for ESCs derivation. According to data from International Embryo Transfer Society (IETS), global production and transfer of IVF bovine embryos increased over 10-fold during the past decade. In 2015, over 60,000 embryos were produced in vitro and approximately 40,000 were transferred globally, contributing to ~ 50% of total transferred embryos [52]. Large international breeding corporations, such as ABS Global, Inc., Semex, and Alta Genetics Inc., as well as specialized suppliers of IVF services, such as TransOva Genetics and L’Alliance Boviteq, have significantly accelerated the commercial usage of IVF in driving genetic improvement in herds [53]. In South America, the extensive application of IVF embryos in the breeding scheme of beef cattle plays a determinant role in rapidly accelerating genetic improvement in herds [52, 54].

Compared with in vivo conceived embryos, the IVF embryos often have compromised developmental potential, particularly in certain domestic species. By using standard or chemically defined culture conditions in combination with different growth factors during oocyte maturation or embryo culture, e.g., colony-stimulating factor, bone morphogenetic protein 15, LIF, natriuretic peptide type C, and/or biologically active small molecules, e.g., 3-isobutylmethylxanthine, 5-aza-2′-deoxycytidine, the efficiency in producing IVF embryos and their developmental potential have been substantially improved [55–59]. Thus, further understanding of oocyte and embryo physiology will assist the development of safer and more efficient IVF systems for producing competent embryos to support the proposed in vitro breeding system.

## Genome-wide sequencing

Single-cell genomic DNA amplification technology has been fully established and can sequence the genome of various species [60–62]. Thus, genome-wide variations of each candidate embryo can be obtained by genome-wide sequencing of one or more cells from the embryo to estimate GEBV. Amplification bias and heterogeneity/uniformity of several commonly used single-cell whole genome amplification kits may have an impact on subsequent SNV calling and copy number variations (CNVs) [60]. However, newly developed direct library construction has addressed these technical limitations [63]. The rapid development of automated and process-based whole-genome sequencing library construction programs have also helped achieve large-scale embryo genetic screening and characterization [64]. Moreover, the well-established protocols for preimplantation genetic screening (PGS) or sex determination utilizing blastomere or trophectoderm biopsies have been used successfully in large-scale commercial dairy cow breeding and propagation without evident adverse effects on subsequent fetal development and postnatal growth. Thus, the well-controlled biopsy of preimplantation embryos will be a safe and valid approach to obtain genomic information from a preimplantation embryo without sacrificing the quality of the tested embryos.

## Genomic selection

Genomic selection is a milestone in animal breeding. Compare to conventional animal genetic selection programs that use individual GEBV, an important feature of our proposed system is to use embryonic GEBV instead. Numerous theoretical breeding studies and applications have confirmed that the GEBV can replace the conventional pedigree-based estimated breeding value entirely as long as the reference population, number of markers, and prediction equation meet the basic requirements [8, 65, 66]. Genomic selection has been widely used in the commercial breeding of animals such as dairy [16, 67] and beef cattle [68], pigs [69, 70], chickens [71–73], and sheep [74]. Following the introduction of genomic selection, the annual genetic improvement of yield traits in American dairy cows has increased by about 50–100% compared to the conventional breeding systems; the progress of some low heritability traits has increased by about 3–4 times [16].

However, the accuracy of genomic selection might decrease as multiple generation selection using the same referene population. At first several generations, our proposed breeding system can still achieve high accuracy of selection as the large reference population and whole genome variations will be used. For later generations, we can add production population phenotypes in the reference population to maintain the accuracy of GS (Fig. 2d). During this selection mating stage, we need to carefully design the mating between male and female embryos to avoid the increase of inbreeding in the population.

As the significant shorter generation interval for this E-to-E breeding system, it is possible that some detrimental mutations would accumulate in the embryo breeding populations before more phenotypes show up. This should be carefully considered when executing the breeding program. Efforts should be taken to reduce the potential damages of harmful mutations by considering all known mutations, and also develop more powerful prediction for these new mutations. Ineed, several algorithms such as SIFT, PolyPhen-2, and CADD, and EVmutation are available to estimate the mutation effects (refs: NBT,2017, Mutation effects predicted from sequence co-variation). More bioinformatic analysis may need to be included in the GS pipeline for novel mutations.

Compared to whole-genome sequencing, chip-based genotyping techniques are limited in detecting insertions/deletions (indels) and CNVs. Therefore, whole-genome sequencing data can yield more informative genetic variations and can further improve the accuracy of genomic selection [10]. Except yield and growth rate, traits such as quality and disease resistance will gain more attention; the need for SNPs will also increase substantially. In addition, developing genome-wide markers is of great significance for maximizing future use of reference populations and data from different reference populations [10, 66]. As the cost of sequencing is drastically reduced, a whole-genome sequencing–based genotyping approach will be an important new development for future genomic selection.

As the number of traits, SNPs, and candidate breeding animals and size of reference population will continue to increase and simultaneous whole-genome sequencing of thousands or more individuals produces massive data, it is possible that computation time will be an important limitation in commercial breeding programs. Continuous optimization will be required for reducing computation expenditure for genomic selection analysis–related processes, such as analysis of massive sequencing data, haplotyping, imputation, and model selection. A robust and scalable data handling and analysis pipeline will be desired for these sequencing data and phenotypic data.

## Conclusions and outlooks

The embryo-stem cell breeding system has significant advantages compared to the conventional breeding system, especially in shortening generation interval, increasing the number of female monotocous offspring, and selection intensity. Taking breeding dairy cows as an example, ideally, our envisioned system is expected to be 30–40 times more efficient in comparison to the conventional system, meaning that 1-year genetic gain of in vitro breeding can be the same as that of 30–40 years of conventional breeding.

The establishment of in vitro germ cell induction, as well as the generation of subsquent embryos and offspring in farm animals, remain the most fundamental challenges for creating an embryo-stem cell breeding system. High-quality and stable ESC lines are prerequisite for achieving in vitro germ cell induction. During the preparation of our manuscript, a recent study reported the efficient derivation and stable propagation of bovine ESCs, and this provides us an unshakeable confidence for constituting the proposed breeding system. However, huge effort remains to be required since high-quality ESCs have not been proven in pigs or other domestic species. As well, even using the recently-reported pluripotent ESCs, the in vitro germ line induction in bovines will be a great challenge. In addition, the improvement and optimization of IVF and genomic selection technologies, highlighting their integration in the embryo-stem cell breeding system, are also needed.

**Fig. 4:**
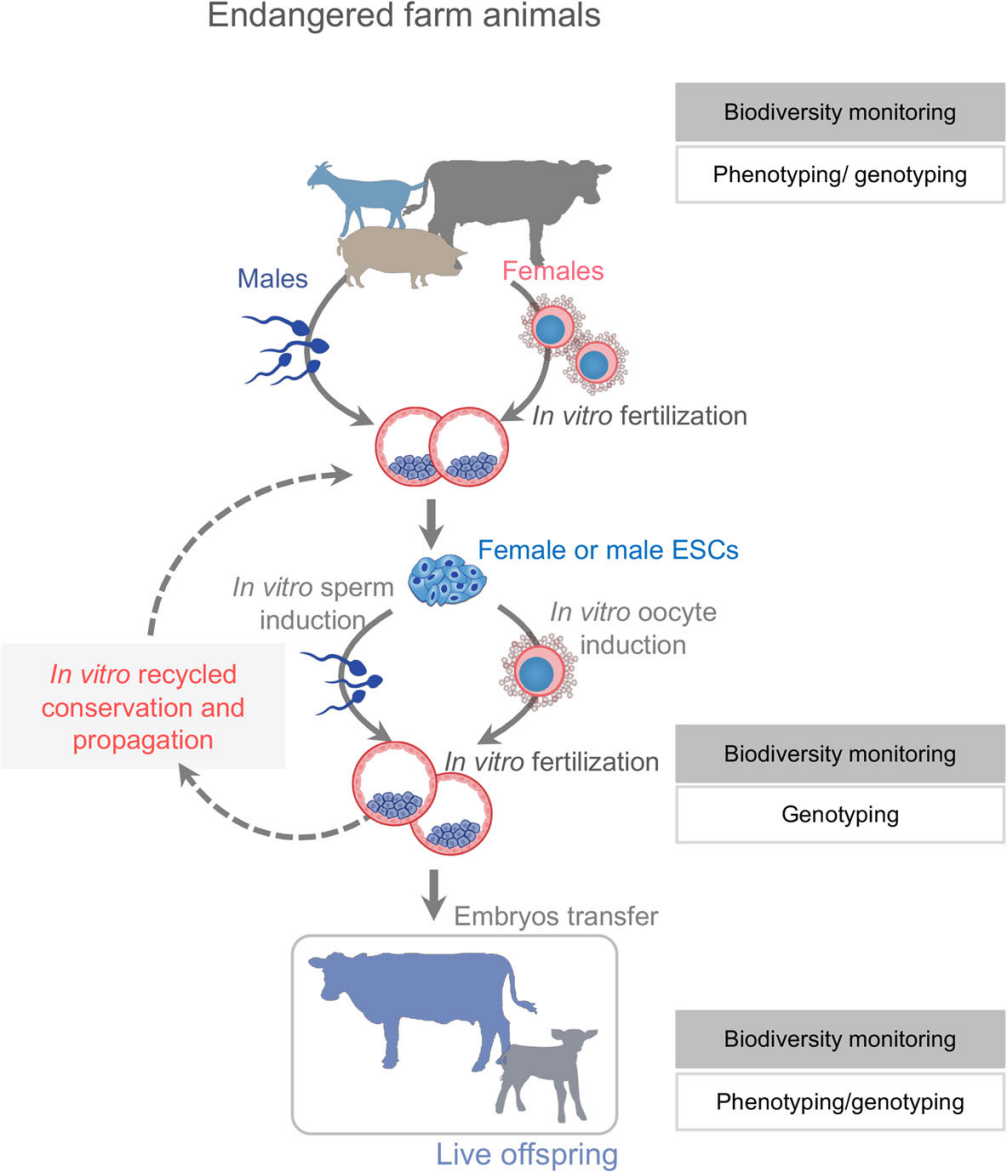
A schematic workflow of the animal embryo-stem cell conservation system**.** The endangered animals under biodiversity monitoring (phenotyping/ genotyping) are used to generate embryos and ESCs sequentially. Population of endangered or rare animal embryos can be quickly expanded through in vitro recycled propagation. Live offspring can be obtained through embryo transfer to recipient of same or relative species/breeds as needed

Farm animal populations harbor numerous genetic variations with phenotypic effects and thus serve as a unique model for understanding the genetic basis of phenotypic diversity. The breeding practice of our system will extend the understanding of genetic basis, e.g. genetic transmission, recombination, and variance under in vitro-reconstituted E-to-E life cycle. Based on our breeding system, one can create an embryo-stem cell conservation system of endangered animals. Many of the endangered or rare species encounter a notable difficulty in propagation due to poor fertility, especially for those with a long generational interval and a small litter size. In our proposed system, the population of these species can be quickly expanded and live offspring can be obtained through embryo transfer as needed (Fig. 4). Lastly, rapid E-to-E life cycle also offers a unique model for studying molecular evolution and artificial selection.

## Abbreviations

ESCs: Embryonic stem cells
GEBV: Genomic estimated breeding values
GS: Genomic selection
iPSCs: Induced pluripotent stem cells
IVF: In vitro fertilization
MAS: Marker-assisted selection
PAGE: Promotion of alleles by genome editing
PGCs: Primordial germ cells
PSCs: Pluripotent stem cells
SNPs: Single-nucleotide polymorphisms
